# Evidence of sex-bias in gene expression in the brain transcriptome of two populations of rainbow trout (*Oncorhynchus mykiss*) with divergent life histories

**DOI:** 10.1371/journal.pone.0193009

**Published:** 2018-02-15

**Authors:** Matthew C. Hale, Garrett J. McKinney, Frank P. Thrower, Krista M. Nichols

**Affiliations:** 1 Department of Biological Sciences, Purdue University, West State Street, West Lafayette, Indiana, United States of America; 2 Department of Biology, Texas Christian University, Fort Worth, Texas, United States of America; 3 School of Aquatic and Fisheries Sciences, University of Washington, Seattle, Washington, United States of America; 4 Ted Stevens Marine Research Institute, Alaska Fisheries Science Center, National Marine Fisheries Service, NOAA, Juneau, Alaska, United States of America; 5 Conservation Biology Division, Northwest Fisheries Science Center, National Marine Fisheries Service, NOAA, Seattle, Washington, United States of America; University of Missouri Columbia, UNITED STATES

## Abstract

Sex-bias in gene expression is a mechanism that can generate phenotypic variance between the sexes, however, relatively little is known about how patterns of sex-bias vary during development, and how variable sex-bias is between different populations. To that end, we measured sex-bias in gene expression in the brain transcriptome of rainbow trout (*Oncorhynchus mykiss*) during the first two years of development. Our sampling included from the fry stage through to when *O*. *mykiss* either migrate to the ocean or remain resident and undergo sexual maturation. Samples came from two F_1_ lines: One from migratory steelhead trout and one from resident rainbow trout. All samples were reared in a common garden environment and RNA sequencing (RNA-seq) was used to estimate patterns of gene expression. A total of 1,716 (4.6% of total) genes showed evidence of sex-bias in gene expression in at least one time point. The majority (96.7%) of sex-biased genes were differentially expressed during the second year of development, indicating that patterns of sex-bias in expression are tied to key developmental events, such as migration and sexual maturation. Mapping of differentially expressed genes to the *O*. *mykiss* genome revealed that the X chromosome is enriched for female upregulated genes, and this may indicate a lack of dosage compensation in rainbow trout. There were many more sex-biased genes in the migratory line than the resident line suggesting differences in patterns of gene expression in the brain between populations subjected to different forces of selection. Overall, our results suggest that there is considerable variation in the extent and identity of genes exhibiting sex-bias during the first two years of life. These differentially expressed genes may be connected to developmental differences between the sexes, and/or between adopting a resident or migratory life history.

## Introduction

Males and females of many species exhibit divergence in phenotypes such as body size, morphology, behavior, and physiology [1–3]. Like other adaptive traits, these phenotypes can be subjected to different selective regimes between environments [4]. Males and females often have different fitness optima based on intraspecific competition or sex-specific selection, which can lead to different degrees of sexual dimorphism within populations [4–8].

Although phenotypic differences between the sexes are common, the same complement of genes is present in both sexes (with the exception of genes located on heterogametic sex chromosomes [9]). As a result, the genetic basis underlying phenotypic sex differences are largely due to sex-biased gene expression, which includes genes that are expressed only in one sex as well as genes that are expressed at different levels between males and females [9–10]. Sex-bias in gene expression provides a mechanism for organisms to produce different adaptive phenotypes using the same genetic background. However, this can also lead to evolutionary constraints when antagonistic selection between the sexes operates on the expression of specific genes [10–12]. Given that large-scale patterns of sex-biased gene expression have been demonstrated in many species [9, 13–17] it seems that sex-biased genes may be common targets of sex specific selection. Indeed, Moghadam [18] found that the W chromosome in chickens has responded to selection for female specific traits in W-linked genes, and similar results have been reported for male specific traits on the Y chromosome in *Drosophila* [19].

In rainbow trout (*Oncorhynchus mykiss*), previous research has found evidence of sex-bias in gene expression [16, 20–21]. These differences include sex determining and sex differentiation genes [20–21] and genes without known sex-specific function [16]. While informative, these studies either focused on candidate genes, or used the 16k cGRASP microarray chip [22], and therefore are limited to predetermined gene sets. RNA-seq methods aim to sequence the majority of transcripts being expressed in a tissue, thereby getting a complete picture of gene expression. In addition, previous studies measuring sex-bias in expression in rainbow trout either sampled the gonads [21], or used whole embryos [16, 20]; however, patterns of sex-bias in gene expression are often tissue specific, limiting their interpretation to the tissue studied [11, 15, 23–25]. The brain is a key component of the brain-pituitary-gonadal axis. As such, expression of genes in the brain may have a profound effect on the processes of sexual maturation and sexual differentiation, as well as being linked to sex-specific behaviors more broadly [26].

We used RNA-seq to evaluate sex-bias in gene expression in the brain of juvenile *O*. *mykiss* during the first two years of development. These samples were derived from two populations within the Sashin Creek river system in South East Alaska that differ in their migratory life history; 1) resident samples from Sashin Lake and 2) migrant individuals from Sashin Creek. These populations have become a model system for answering questions concerning the genetic basis of migration [27–31] and resident and migrant life histories have demonstrated differences in growth rate, age at maturation, life history type, and seawater survival. In addition, there are significant differences between the sexes in several adaptive phenotypes such as weight, growth rate, and osmoregulatory capacity [27–28, 32]. However, no previous study has investigated patterns of sex-bias in gene expression in these populations. To that end, two existing RNA-seq datasets from Sashin Creek [30–31] offer an opportunity to examine sex-specific differences in gene expression during the first two years of development. These datasets were previously used to examine patterns of differential gene expression between migratory and non-migratory rainbow trout eco-types [30–31]. In this study, we combined and reanalyzed these data to examine sex-bias in gene expression to answer two questions: 1) to evaluate broad scale patterns of sex-bias in gene expression over the course of juvenile development, and 2) to determine if there are differences in sex-biased gene expression between migratory and resident populations of *O*. *mykiss* which face different selection regimes.

## Materials and methods

### Study system

All methods involving live Rainbow Trout were approved by Purdue University’s IACUC (protocol number 06–033). Before sample extraction all fish were anesthetized (parents of cross) or euthanized (for brain extraction) using either clove oil or tricaine methanesulphonate (details presented below). Samples for study were generated from crosses using migratory and resident adult *O*. *mykiss* sampled from natural populations in Sashin Creek and Sashin Lake, Alaska, respectively. Mature adults were collected in May 2010. Migratory samples were collected from a weir that captures returning adults as they return to Sashin Creek to spawn and resident samples were collected by fyke nets from Sashin Lake. At collection, fish were anesthetized with clove oil (25 mg/L), or tricaine methanesulphonate (50 mg/L: MS-222; Argent Chemicals, Redmond, WA), and gametes were expressed into individual bags by light pressure on the abdominal cavity. Gametes were stored for a maximum of 24 h before being used to generate crosses. For this study, eight full-sibling crosses were initiated on 27 May 2010: four families were generated from mating of four separate anadromous female with four separate anadromous male (A x A cross type), and four families were generated by crossing four resident females with four resident males (R x R cross type). Embryos were reared at ambient creek temperatures in recirculating stack incubators in the dark. Once the fish reached swim-up (utilization of yolk) each family was thinned to equal densities, and reared in individual outdoor vertical raceways (flow through water came from Sashin Creek, and samples were kept under natural photoperiod). At one year of age, approximately 50 fish from each family were combined into larger vertical raceways. Flow through water came from Sashin Creek, and fish were kept under natural photoperiod.

Five offspring per family were sampled at each of five time points from four months after hatch until two years of age. Two-years of age is the time point when smoltification (salt water adaptation) peaks in this population of *O*. *mykiss*. These time points were: four months after hatch (October 2010), eight months after hatch (February 2011), twelve months after hatch (June 2011), twenty months after hatch (February 2012), and twenty-four months after hatch (June 2012). At all time points, fish were euthanized with a lethal dose of MS-222 (50 mg/L) and brains and liver (for DNA extraction) were immediately dissected and placed in RNAlater (Applied Biosystems, Foster City, CA). All samples were stored at -80°C. DNA was extracted from liver samples using phenol-chloroform [33] for the purpose of sexing individuals with the *Omy-Y1* marker [34] as described by Hecht et al. [28].

### RNA extraction and sequencing

Two males and two females from each cross type were chosen for RNA-seq for the first four time points and eight females and seven males were chosen for RNA-seq at the last time point (4 of each sex for the R x R cross and 3 males and 4 females from the A x A cross) for a total of forty-seven individuals [30–31] RNA was extracted using either Trizol (Ambion, Foster City, CA) following the manufacturer’s protocol (time points one to three), or a combination of Trizol and RNeasy columns (time points four and five; Qiagen, Valencia, CA). At all time points, whole brains were homogenized and RNA extracted. Sample library preparation and sequencing was conducted at the Purdue University Genomics Core Facility. Samples were analyzed for concentration and quality using a Nanodrop (Thermo Scientific, Waltham, MA). Column cleanup was performed on samples with low 260/230 or 260/280 absorbance ratios (YM-50 column, Amicon). Final quality control was conducted on a 2100 Bioanalyzer using a NRA 6000 Nano chip (Agilent Technologies). A minimum RIN score of 8.0 was required for library construction. Libraries were prepared using TruSeq RNA sample preparation kits (Illumina, San Diego, CA), and nine lanes of sequencing was conducted on an Illumina Hi-Seq 2000 (v3 chemistry) using 100bp paired-end reads.

### Gene expression

The first year samples in this study were previously used to produce a transcriptome assembly [30]. Quality-filtered sequence reads from all samples were aligned to the assembly [30–31]. To obtain a conservative estimate of gene expression, (i.e. to remove allelic variants, or isotigs) quantification of read counts was done at the gene level (annotation of contigs within components were consistently annotated as the same gene; see [30] for details). Sequence reads for each individual were aligned to the transcriptome assembly using RNA-seq by Expectation Maximization (RSEM) [35] with the default settings and transcript-to-gene map option to obtain component level read counts. Read counts for genes were imported into edgeR [36] for statistical analysis. Raw read counts were converted to counts per million (cpm) to normalize for differences in library size. Genes were required to pass a count threshold where 3 out of 8 individuals in the first four time points, and 7 out of 15 individuals in the last time point (i.e. one less than half) were required to have at least 1 cpm. Genes that did not pass this threshold for at least one time point were removed from analysis. Remaining gene read counts were then normalized using trimmed mean of m-values (TMM) normalization to account for differences in library composition [37] Normalized counts were analyzed for differences in expression by combining all variables into a three-way factor using the following model: Y_ijk_ = μ + S_i_ + A_j_ + T_k_ + S_i_A_j_T_k_ + E_ijk_ where Y_ijk_ is the log_2_ comparison between samples for sex _i_; cross _j_, and time point _k_: μ is the mean, T is the time point of the sample (i.e. four months, twenty months, etc.), A is the cross of the sample (A x A or R x R), S is the sex (male or female), SAT is the three way interaction, and E is the error. Contrasts of interest were between individuals of different sex (male and female) but were from the same cross and the same time point. All models were run in edgeR [36]. Log_2_ fold-change, p value, and false discovery rate (FDR, alpha = 0.05; [38]) corrected p values were used for further analysis. Log_2_ fold-change and p value were used to test for significance in biological function and pathway analysis. Annotation of contigs from genes that passed count thresholds was achieved using the ENSEMBL [39] human (assembly GRCh37, release 73) and zebrafish (assembly Zv9, release 73) as described previously [30].

### Biological function and pathway analysis

Ingenuity Pathway Analysis (IPA) software (Ingenuity Systems, Redwood City, CA) was used to identify biofunctions and canonical pathways that were enriched for sex-biased genes. Data (annotations, log_2_ fold-change, and p values from EdgeR analysis) were uploaded into IPA using the human or zebrafish annotations from ENSEMBL, as described above. Thresholds within IPA for considering a component differentially expressed were set at p <0.01 and fold-change>1.8. Since IPA is not testing the significance of genes as single entities, but rather their significance when combined in networks with other genes, a multiple testing correction was not done prior to importing the data into IPA to prevent inflated type-II error, which is of particular concern when sample size is low and variability is high [40]. Since the downstream pathway analyses are highly dependent upon the initial list of differentially expressed genes we instead focused our analysis on pathways and biofunctions that were identified in IPA as enriched. Enriched pathways required both a Fisher’s exact test (p <0.05), following the approach used by [30–31], and a regulation Z-score either higher than 2 or lower than -2.

### Gene mapping

All sex-biased genes were mapped to a draft version of the *O*. *mykiss* genome [41] using Blastn. All mapped genes had a minimum e-value of 1e-10, a maximum number of 3 mismatches, and a minimum alignment length of 100 nucleotides. Each mapped gene was allowed to align to one region of the genome. Any differentially expressed genes that mapped to multiple regions of the same chromosome, or different chromosomes were removed. A chi-square approach (alpha < 0.05) was used to test if any chromosome was enriched for more female or male biased genes than expected by chance.

### Availability of supporting data

Raw Illumina sequence data used in this manuscript have been deposited in the GenBank Short Read Archive (SRA). The accession numbers are SRP050380 for the first year samples, and PRJNA269115 for the second year samples. The transcriptome assembly used for gene expression in this study can be found in the Data Dryad (http://dx.doi.org/10.5061/dryad.ch264).

## Results

A total of 1,350,177,271 (95% of total reads) Illumina paired-end quality filtered reads were used for analysis. These reads were mapped to a previously published *O*. *mykiss* transcriptome from the same population [30]. Genes that failed to meet the minimum count threshold of at least 3 samples having a cpm >1 for the first four time points, or 7 samples having a cpm > 1 for the fifth time point (i.e. one less than half) were removed, leaving a total of 36,929 genes for which we evaluated differential gene expression. The number of paired-reads that aligned to the transcriptome per individual varied from 7.03 million to 30.31 million [30,31]. In total, 22,410 genes were annotated with the ENSEMBL human and zebrafish databases for use in IPA. When the same annotation exists for multiple genes, IPA keeps the gene with the greatest fold change. After removal of duplicate annotations, 12,326 genes were retained and analyzed by IPA.

### Differential gene expression

**Fig 1:**
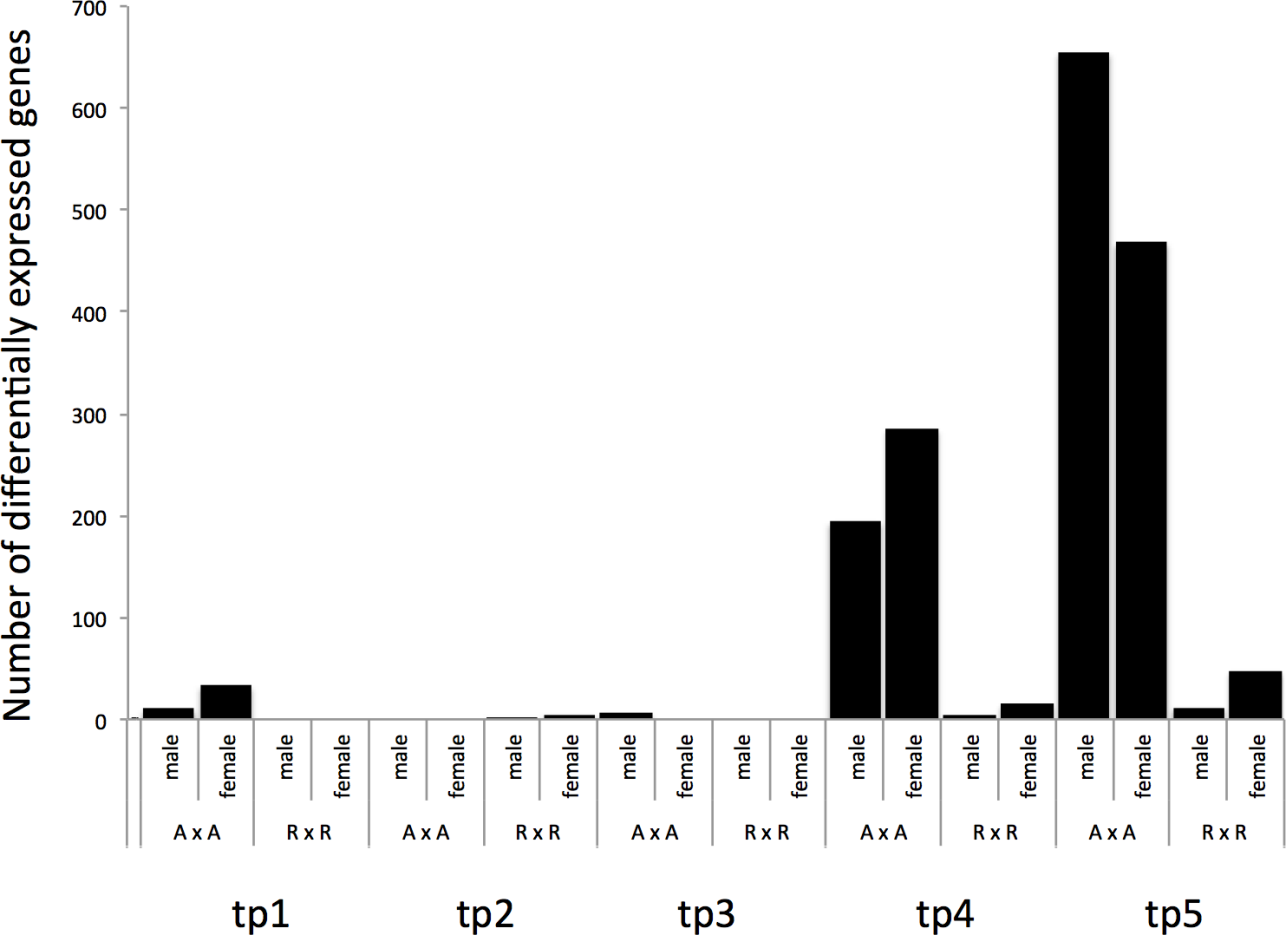
Number of genes that were differentially expressed (DE) between males and females for each time point and cross type. A x A samples were produced by crossing anadromous parents that returned to Sashin Creek to spawn. R x R samples were produced by crossing resident parents from Sashin Lake.

**Fig 2:**
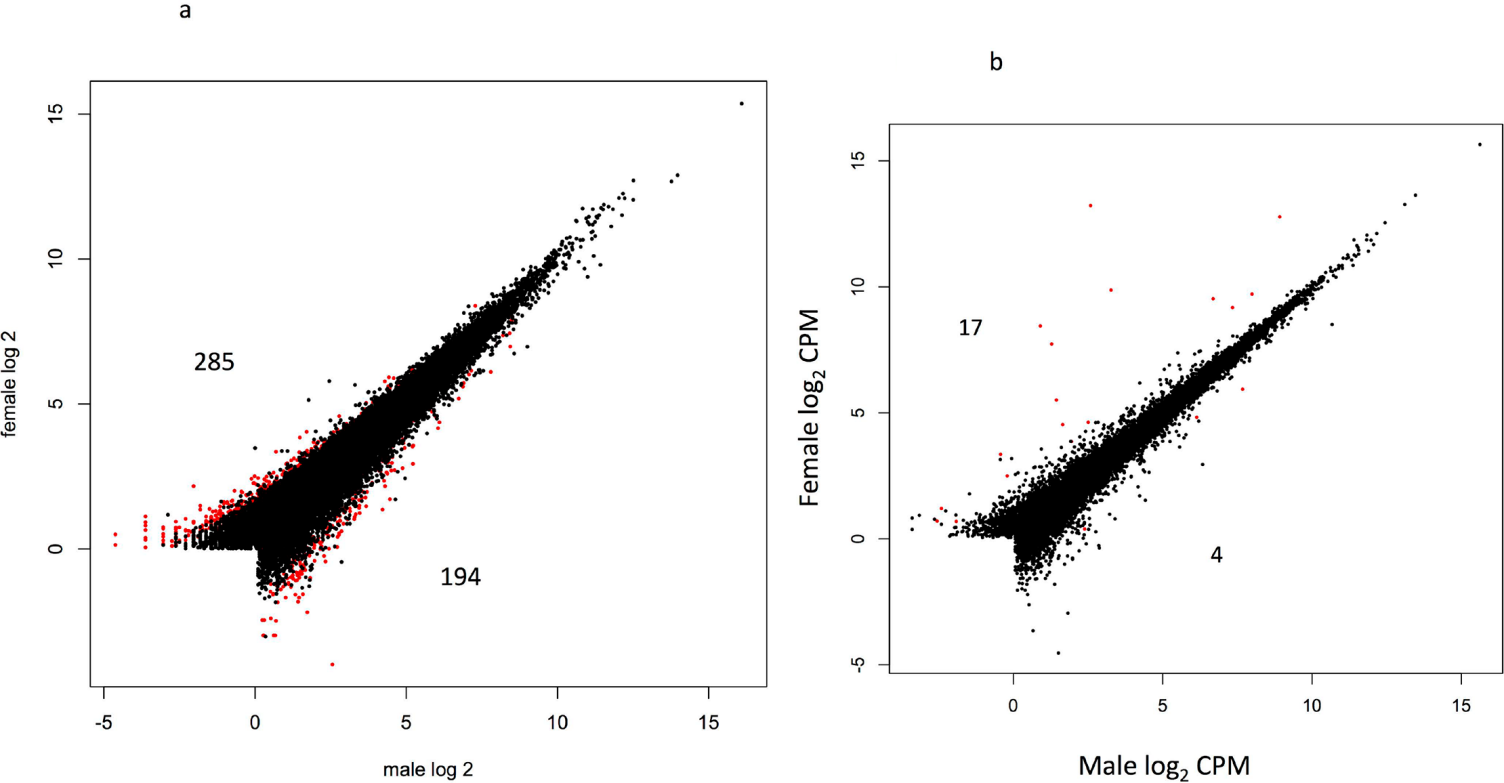
Relative gene expression in males and females sampled in February of the second year (time point 4) from both the A x A cross (Fig 2A), and the R x R cross (Fig 2B). Red points represent differentially expressed genes between the sexes (FDR correct p = <0.05); points in black represent genes that were not differentially expressed.

**Fig 3:**
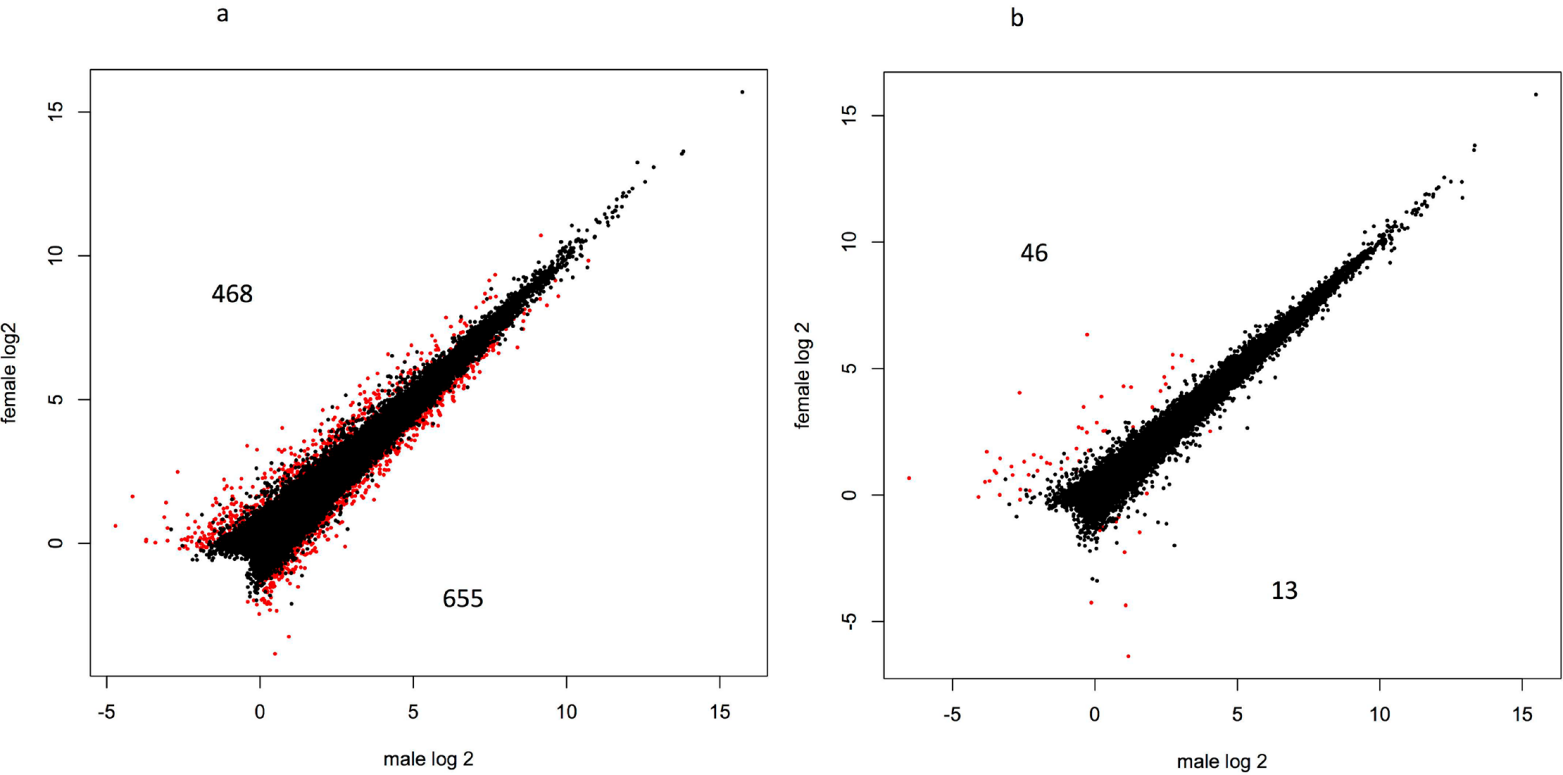
Relative gene expression in males and females sampled in June of the second year (time point 5) from both the A x A cross (Fig 3A), and the R x R cross (Fig 3B). Data points in red represent differentially expressed genes between the sexes (FDR correct p = <0.05), data points in black represent genes that were not differentially expressed.

Sex-bias in gene expression was inferred by applying GLM approaches within each cross type and within each time point. A false discovery rate (FDR) of 5% was used as a threshold for significance in all tests. A total of 1,716 (7.2%) genes were differentially expressed between the sexes in at least one time point (Fig 1), however only 42 of these were shared between multiple time points (S1 Table shows details of expression for all genes; S2 Table shows annotated sex-biased genes). A total of 58 genes were differentially expressed between males and females during the first year of life. Of which, 45 showed sex-bias in expression soon after swim up in the A x A samples, and six genes were differentially expressed between the sexes at eight months post hatch in R x R samples and 12 months post hatch in the A x A samples. No other contrast showed evidence of sex-bias in gene expression in the first year of age, and none of the differentially expressed genes were shared between the first year and second year samples. Many more genes (1,663) showed differential expression in the second year of life (Fig 1). However, there were differences in the number of sex-biased genes between contrasts with more genes showing sex-bias in expression in the A x A samples compared to the R x R samples. For example, there were 479 sex-biased genes in the 20 month old A x A samples, and only 19 sex-biased genes in the R x R samples from the same time point. Similar results were found in 24 month old samples with 1,123 genes showing differential expression between the sexes in the A x A samples, and 59 genes were sex-biased in the R x R samples. No genes showed sex-bias in both cross types in 20 month old samples, and two genes were differentially expressed between the sexes in both cross types in the 24 month old samples. Sixteen genes showed sex-bias in expression in both the 20 and 24 month old samples in the A x A cross. Of which, nine genes were consistently upregulated in females, four genes were consistently upregulated in males, and three genes were upregulated in females in the 20 month old samples and were upregulated in males in the 24 month old samples. Although there was little difference in the overall number of sex-bias genes upregulated in males and females (883 versus 857 respectively) there were differences between contrasts. For example, in samples from time point 4 (20 month-old samples) time point in the A x A samples 285 sex-bias genes were upregulated in females compared to 194 sex-bias genes upregulated in males (Fig 2). However, the proportion was reversed at two years of age (time point 5) with 655 sex-bias genes upregulated in males from the A x A contrast, compared to 468 sex-biased genes upregulated in females (Fig 3).

### Biofunction analysis

Functional analysis revealed 165 biofunctions that were enriched for sex-biased genes across contrasts (S3 Table). Of these, 38 were enriched in samples from the first year of life, and two biofunctions (Organismal Death, and Hypertrophy of Heart Cells) were enriched for sex-biased genes in multiple contrasts. The number of enriched biofunctions varied from 23 in A x A four month samples, to zero in R x R 12 month samples. A total of 133 biofunctions were enriched for sex-biased genes in the second year of life. Of these 38 biofunctions were shared between multiple contrasts. Five biofunctions (Organismal Death, Activation of Cells, Development of Connective Tissue, Hypertrophy of Heart Cells, and Transport of Molecules) were enriched for sex-biased genes in multiple contrasts between year one and year two. The most commonly enriched biofunctions were Organismal Death (five contrasts), Proliferation of Cells (four contrasts), and Cell Movement (three contrasts). An additional 31 biofunctions were enriched in two contrasts, and 131 biofunctions were enriched in one contrast. This suggests limited temporal overlap of enriched biofunctions, and that patterns of gene expression are changing during the first two years of development. Across all time points, more biofunctions were enriched in A x A samples (152 different biofunctions) compared to R x R samples (29 different biofunctions).

### Pathway analysis

A total of 194 pathways (between 27 and 92 pathways within each time point) were enriched for sex biased genes in at least one time point (S4 Table). Two pathways (Visual Cycle, and Calcium Signaling) were altered in all contrasts, and an additional nine pathways were altered in 9 out of 10 contrasts (see Table 1). Samples from the first year showed 128 pathways that were enriched for sex-biased genes of which five pathways (Calcium Signaling, The Visual Cycle, Agranulocyte Adhesion and Diapedesis, Phototransduction Pathway, and Retinoate Biosynthesis I) were enriched in all contrasts suggesting many sex-biased genes are involved with photoreception and vision. A total of 93 pathways were enriched in either one or two contrasts suggesting variation in patterns of gene expression during the first year. A total of 147 pathways were enriched in the second year samples of which 80 were also enriched for sex-biased genes in at least one contrasts in samples from the first year. Twelve canonical pathways were enriched in all four contrasts from the second year (Calcium Signaling, The Visual Cycle, Tight Junction Signaling, Cellular Effects of Sildenafil, Epithelial Adherens Junction Signaling, Sertoli Cell Junction Signaling, Maturity Onset Diabetes of Young Signaling, ILK Signaling, Leukocyte Extravasation Signaling, FXR/RXR Activation, Clathrin-mediated Endocytosis Signaling, and Germ Cell-Sertoli Cell Junction Signaling) suggesting enrichment for sex-biased genes in pathways involved with cell signaling, and sexual development. Like the first year, many pathways were enriched only for one (78 pathways) or two (35 pathways) contrasts, again suggesting that patterns of sex-bias in gene expression change during the second year of life. All altered pathways are shown in S4 Table.

**Table 1:** Canonical pathways that were enriched for sex-biased genes in ten or nine contrasts. A full list of all enriched canonical pathways, and the associated molecules within the pathway are given in S3 Table. p-values for each pathway are reported for all datasets. Time point 1 = 4 months post hatch, time point 2 = 8 months post hatch, time point 3 = 12 months post hatch, time point 4 = 20 months post hatch, time point 5 = 24 months post hatch. Both cross types (A x A and R x R) are shown.

Canonical pathway	1 A x A	1 R x R	2 A x A	2 R x R	3 A x A	3 R x R	4 A x A	4 R x R	5 A x A	5 R x R
Calcium Signaling	0.000	0.019	0.000	0.010	0.000	0.000	0.000	0.000	0.000	0.000
The Visual Cycle	0.003	0.000	0.001	0.000	0.009	0.036	0.001	0.000	0.002	0.036
Tight Junction Signaling	0.011		0.000	0.001	0.000	0.000	0.001	0.000	0.001	0.000
Cellular Effects of Sildenafil (Viagra)	0.003		0.000	0.009	0.000	0.000	0.000	0.000	0.000	0.005
Agranulocyte Adhesion and Diapedesis	0.001	0.001	0.011	0.000	0.001	0.000		0.000	0.000	0.000
Phototransduction Pathway	0.000	0.002	0.000	0.000	0.000	0.004	0.000	0.000	0.000	
Retinoate Biosynthesis I	0.002	0.009	0.002	0.011	0.015	0.026	0.033	0.001	0.035	
Epithelial Adherens Junction Signaling	0.003		0.002	0.047	0.002	0.001	0.004	0.000	0.000	0.010
Sertoli Cell-Sertoli Cell Junction Signaling		0.006	0.050	0.001	0.000	0.010	0.000	0.002	0.002	0.003

### Gene mapping

A total of 1,071 (62.4%) sex-biased genes were mapped to the *O*. *mykiss* genome. The number of mapped sex-biased genes varied from 67 for Omy5 to 0 for Omy25. A total of 512 mapped sex-biased genes were upregulated in males and 559 were upregulated in females. Only the X chromosome had a significant difference in the number of male and female upregulated genes (9 male, 23 female; χ^2^ = 6.125, p = 0.013) than expected by chance (although Omy6 is notable (19 male, 33 female; χ^2^ = 3.769, p = 0.052)). A total of 831 mapped sex-biased genes were annotated. Table 2 shows those annotated sex-biased genes that mapped to the X chromosome. All annotated mapped sex-biased genes are reported in S5 Table.

**Table 2:** Annotated sex-biased genes that mapped to the X chromosome. Genes with a negative log_2_ fold change were upregulated in females and genes with a positive log_2_ fold change were upregulated in males. A full list of all mapped sex-biased genes are shown in S4 Table.

component	Log_2_ fold change	Accession number	Gene ID	Gene description
comp630510	-4.756	ENST00000394997	HIF1A	hypoxia inducible factor 1, alpha subunit (basic helix-loop-helix transcription factor)
comp650683	-3.477	ENST00000601328	RXRA	retinoid X receptor, alpha
comp631583	-2.416	ENST00000309989	DAB2IP	DAB2 interacting protein
comp648565	-2.227	ENST00000298386	RXFP2	relaxin/insulin-like family peptide receptor 2
comp647634	-2.171	ENST00000498275	ZDHHC23	zinc finger, DHHC-type containing 23
comp638820	-1.909	ENST00000541405	CKAP2L	cytoskeleton associated protein 2-like
comp630811	-1.765	ENST00000575648	WBSCR27	Williams Beuren syndrome chromosome region 27
comp618719	-1.509	ENST00000509430	GFM2	G elongation factor, mitochondrial 2
comp618268	-1.501	ENST00000594292	CCNB3	cyclin B3
comp652873	-1.291	ENST00000305877	BCR	breakpoint cluster region
comp643785	-1.186	ENST00000583356	TANC2	tetratricopeptide repeat, ankyrin repeat and coiled-coil containing 2
comp600566	-1.066	ENST00000394422	UTP14A	UTP14, U3 small nucleolar ribonucleoprotein, homolog A (yeast)
comp653920	-1.037	ENST00000328252	PAPPA	pregnancy-associated plasma protein A, pappalysin 1
comp652184	-1.014	ENST00000342694	NPR2	natriuretic peptide receptor B/guanylate cyclase B (atrionatriuretic peptide receptor B)
comp651855	-0.911	ENST00000218006	GUCY2F	guanylate cyclase 2F, retinal
comp642890	-0.896	ENST00000354268	SMARCAD1	SWI/SNF-related, matrix-associated actin-dependent regulator of chromatin, subfamily a, containing DEAD/H box 1
comp649713	-0.651	ENST00000388824	MEX3D	mex-3 homolog D (C. elegans)
comp646344	0.686	ENST00000301894	NRXN2	neurexin 2
comp640732	1.201	ENST00000393229	NTNG2	netrin G2
comp644924	1.494	ENST00000287538	ZIC3	Zic family member 3
comp552267	2.106	ENST00000263413	C6	complement component 6
comp438713	2.295	ENSDART00000015629	stxbp1a	syntaxin binding protein 1a
comp633675	2.8	ENST00000392870	GRK5	G protein-coupled receptor kinase 5

## Discussion

Many studies in a variety of species have found evidence of sex-bias in gene expression. However, few studies have looked at how sex-bias in expression varies over ontogeny, or how sex-bias in expression varies between different populations of a species (but see 13, 17). In this study, we find two notable patterns in the brain transcriptome of rainbow trout: 1) more genes show sex-bias in expression during the second year of life, just prior to an important developmental life history transition; and 2) there is variation in the extent of sex-bias in gene expression between adjacent populations with many more genes showing sex-bias in the anadromous line than the resident line. Herein, we discuss these findings and their implications for the developmental life history transitions and divergence between alternative migratory tactics in salmonids—that is, either delaying sexual maturation in order to migrate to the ocean before returning to their natal streams to spawn, or staying resident and undergoing earlier sexual maturation [42–43].

### Patterns of sex-bias in gene expression

Previous studies have reported an increase in the number of sex-bias genes during sexual maturation. This is not surprising, as the development of secondary sexual characteristics requires the production of different transcripts in males and females (e.g. 13). The gonads frequently show high levels of sex-bias in gene expression due to their roles in gametogenesis, sexual differentiation, and sexual development [17, 25, 44]. However, multiple studies have also shown that the brain exhibits sex-bias in gene expression [13, 24, 25, 45] albeit to a lesser extent than the gonads. Sex-bias in gene expression in the brain could suggest two non mutually-exclusive processes: 1) sex-bias in gene expression could initiate variation in the secretion of hormones from the pituitary, which in turn regulates the development of secondary sexual characteristics, and 2) differences in gene expression could be a result of differences in neurogenesis and brain development in males and females. Teasing apart these processes is difficult. The number of genes showing sex-bias in expression in this study increased when some males had completed sexual maturation (i.e. expressed milt, which was observed in samples of 24 months of age). This might suggest support for the first hypothesis, as none of the females were sexually mature. However, both sexes showed upregulation of genes at the 24 month time point (655 male upregulated genes and 468 female upregulated genes). As none of the females were sexually mature, the large number of female upregulated genes suggests that sex-bias in expression may be the result of differences in neurogenesis and brain development between males and females. As already mentioned, both processes are undoubtedly involved in explaining the identity of genes that exhibited sex-bias as although some had obvious function to male gametogenesis, many more are involved in brain development (discussed below).

### Differential gene expression and differences in brain development

Previous studies have yielded a number of candidate sex determination and sex differentiation genes in *O*. *mykiss* [20, 21]. Here we found evidence of sex-bias in gene expression in 11 candidate genes, nine of which have a role in brain development and neuronal proliferation (*BIRC5*, *BMP7*, *CHD1*, *GPHB5*, *LHX9*, *GATA2*, *PAX2*, *TBX1* and *WT1*) [46–55]. Differential expression of these candidate genes may be linked to fundamental differences in brain development between males and females [56]. These differences may be due to genes serving different roles in the development of male and female brains, or that temporal patterns of brain development differ between the sexes. Studies in mice and zebrafish found a higher rate of cell death in the brains of males compared to females [57–58]. The biofunction cell death is altered in A x A samples from 24 month old samples and is enriched for genes upregulated in males (Z score = 2.198, p < 0.001) which could suggest that cell turnover is higher in male brains than female brains. However, males are much more likely to undergo sexual maturation at two years of age (i.e., the last sampled time point) and remain resident than females. So perhaps, the enrichment of male-biased genes that make up the biofunction cell death may be connected to sexual maturation.

**Fig 4:**
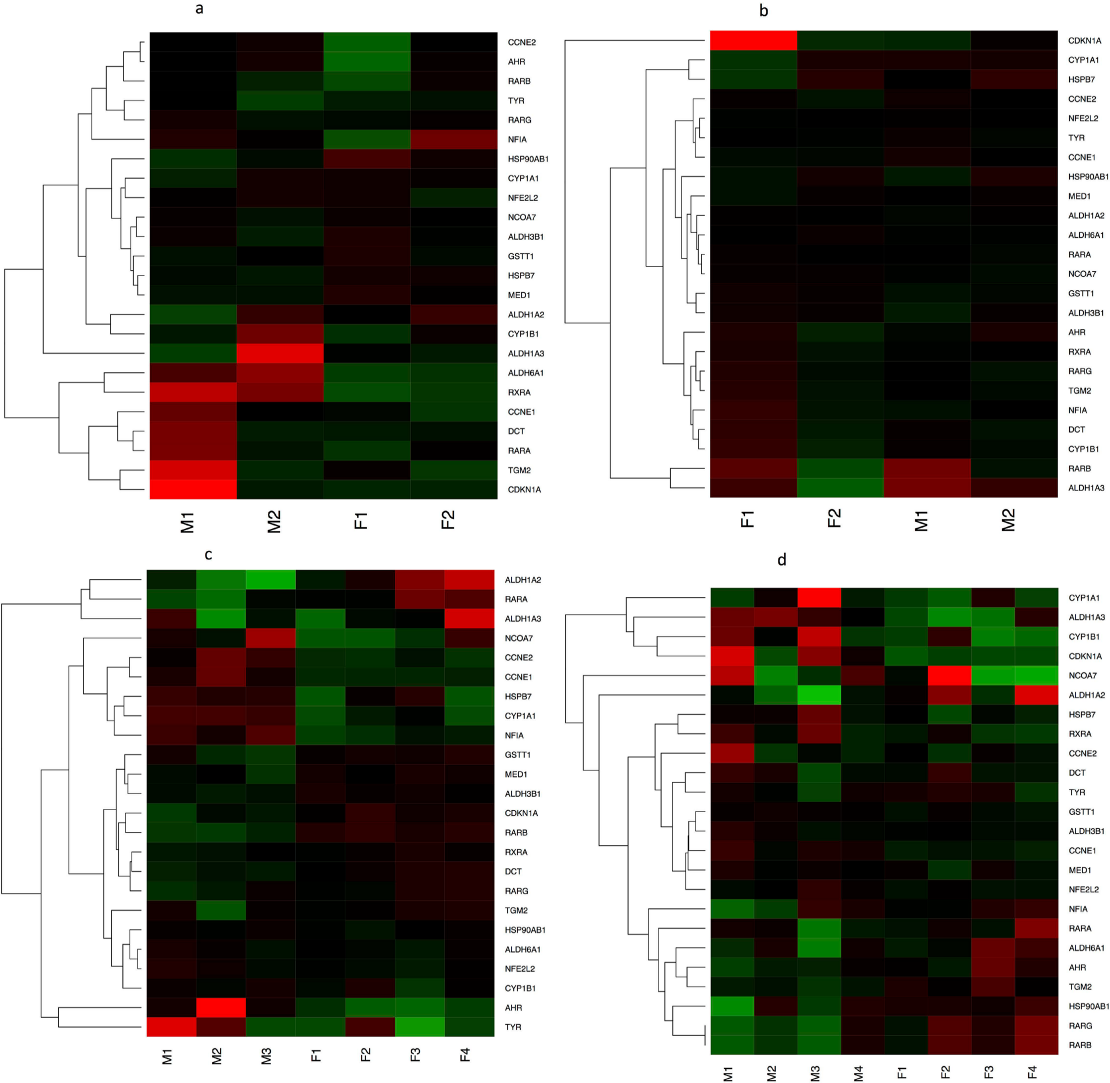
Heat map of log_2_ fold-change of gene expression compared to average expression within treatment for genes involved in the Ahr signaling pathway. Fig 4A shows samples from the A x A cross at time point 4, Fig 4B shows samples from the R x R cross at time point 4, Fig 4C shows samples from the A x A cross at time point 5, and Fig 4D shows samples from the R x R cross at time point 5. Genes in green were upregulated compared to average while genes in red were down regulated compared to average. Genes in black were expressed at similar levels to average.

There were several biological pathways related to reproduction and sexual maturation that showed enrichment for differentially regulated genes in this study. These pathways included aryl hydrocarbon receptor (AhR) signaling, role of janus kinase 2 (JAK2) in hormone-like cytokine signaling, relaxin signaling, sertoli cell—sertoli cell junction signaling, germ cell—sertoli cell signaling, and sperm motility. AhR signaling was altered between males and females for both cross types at four, eight and 24 months old, suggesting genes within the AhR signaling pathway are differentially expressed between the sexes through the first two years of development (see Fig 4A–4D). The AhR pathway is involved in the formation and development of the anteroventral periventricular nucleus (AVPV) [59], a sexually dimorphic structure in the hypothalamus whose functions include modulation of gonadotropins such as luteinizing hormone (LH) [59–61]. LH production increases in salmonids in the lead up to spawning [62–63], stimulating gametogenesis and gamete release [64]. Therefore structures (like the AVPV) that modulate the release of LH are important in sexual differentiation. Although our understanding of the AVPV and its role in sexual maturation has been determined by experiments on mammals, the AVPV is present in fish [65], and there is evidence that the role of the AhR pathway in GABA neurogenesis is conserved across taxa [59]. Therefore, many of the sex-biased genes and altered pathways in rainbow trout in this study may be connected to differences in brain development between males and females.

### Mapping of sex-biased genes

All salmonids have a XX/XY system of sex determination [66]. However, the sex determining gene *sdY* (sexually dimorphic on the Y-chromosome) is located on different chromosomes in different species of salmonid [67–69]. The only chromosome that was enriched for sex-biased genes was the X chromosome (enriched for genes that were upregulated in females). Many previous studies also report enrichment of sex-biased genes on the homogametic sex chromosome in a wide variety of taxa [45, 9, 15, 70]. However, whether the homogametic or heterogametic sex shows upregulation of sex-bias genes on the homogametic chromosome varies, and may be informative with respect to dosage compensation. For example, studies in birds report an upregulation of sex-bias genes on the homogametic sex chromosome in the homogametic sex, and therefore a lack (or reduction) of dosage compensation [11, 71–72]. In contrast, efficient dosage compensation should equalize expression of genes on the homogametic sex chromosome. Here we find enrichment of female sex-biased genes on the rainbow trout X chromosome suggesting either a lack, or a reduction in dosage compensation in rainbow trout. Studies in threespine stickleback (*Gasterosteus aculeatus*) also found enrichment of female-biased genes on the X chromosome [73] however they found that female-biased genes clustered on one region of the nascent X. Leder et al [73] reasoned that this female-biased expression might be due to a lack of dosage compensation, and that the stickleback X is in the early stages of sex chromosome evolution. It is tempting to make similar conclusions regarding dosage compensation in rainbow trout, especially as sex-bias genes on the rainbow trout X do not have obvious annotations to functions linked to sexual development and sexual maturation (see Table 2). Over time, it is hypothesized that genes with sexual function will localize to the sex chromosomes, leading to a reduction in recombination between the proto X and the proto Y chromosome [74]. Moreover, salmonid sex chromosomes are very similar in size and gene content further supporting a recent placement of the sex determining region on the proto Y chromosome [66]. However, our mapping efforts should be interpreted with caution. The draft version of the *O*. *mykiss* genome that was used to map sex-biased genes is not complete [41]. It is possible that the apparent enrichment of female-biased genes on the X chromosome is an artifact of the unfinished nature of the genome. Further completion of the *O*. *mykiss* genome, together with further studies of sex-bias in gene expression from other tissues, and populations of *O*. *mykiss* will increase our understanding of patterns of dosage compensation in rainbow trout.

### Population specific regulation of sex-bias in expression

The second aim of our study was to examine levels of sex-bias in gene expression between two subpopulations of *O*. *mykiss* with divergent life histories. Previous studies have shown that different selection pressure can have profound effects on patterns and the extent of sex-bias in gene expression [18, 75]. The study herein found that samples derived from anadromous parents (A x A cross) had many more genes showing sex-bias in gene expression than samples from those offspring from resident parents (R x R cross). Male salmonids are known to mature at a younger age than females [43]. In this study, all resident males produced from anadromous parents (A x A cross) were actively expressing gametes whereas none of the male smolts, or females had reached sexual maturity. Therefore, the upregulation of male-biased genes in offspring from anadromous parents at this time point may be due to active sexual function in males. However, why this pattern was not mirrored in the R x R cross (12 genes upregulated in males, 47 in females) from the same time point is difficult to explain, as both crosses contained sexually mature males. Perhaps, female offspring from the resident population are beginning the process of sexual maturation, and so are at a similar stage of development to sexually mature males from the resident population. By contrast, offspring from the anadromous population maybe more variable in their timing of sexual maturation. Smoltification (the physiological transition leading to migration to the sea) is highly heritable in this population [27, 32]; moreover, although both migrant and resident fish can produce migrants, migrants produced from resident parents have lower hypo-osmotic capability and reduced ocean survival relative to conspecifics with anadromous parents [76]. Therefore, it seems likely that female offspring produced from anadromous parents (A x A cross) are more predisposed to migrate than female offspring produced from resident parents (R x R cross). The decision to migrate is complex, and requires the coordinated expression of multiple genes, and the upregulation of many hormones [77]. It is possible that the ability for the brain transcriptome to respond correctly to environmental cues and prepare an individual for migration has been compromised in the resident population. There is hypothesized to be selection against the production of migrants in the Sashin Lake (resident) population, because migrants that leave the lake cannot return to spawn [29]. While the loss of coordinated gene expression in the relatively short time since the founding of the resident population (1920s) seems unlikely, studies in multiple breeds of chicken selected for female fecundity traits (layers) have found similar patterns of female biased expression in genes located on the W chromosome, suggesting that selection can operate in a short period of time to alter patterns of gene expression between the sexes [18]. Some genes with functions connected to smoltification such as thyroid hormone responsive (*THRSP*) and somatostatin receptor 1(*SSTR1*) showed sex-bias in expression at the point of smoltification (24 months) in the R x R and A x A cross respectively. Presumably, individuals that upregulate genes connected to smoltification would be better adapted to the demands of seawater, and are more likely to successfully return, further supporting the influence sex has on the decision to migrate in salmonids.

## Conclusions

This study finds two notable patterns concerning sex bias in gene expression in rainbow trout: 1) That many more genes show sex bias during the second year of life (both prior to and during an important life history decision), and 2) that there is variation in the level of gene expression between populations that are subject to different selection regimes. Multiple studies have found that patterns of gene expression within a tissue vary during development, especially during sexual maturation. The brain is a key component of several hormonal axes including axes that regulate both smoltification (and therefore migration) and sexual maturation. The lack of sex bias in the resident population suggests that males and female are on similar developmental trajectories (presumably) to undergo sexual maturation and remain resident. It is likely that life history trajectory is more conserved in the resident than the anadromous population as the resident fish are subjected to strong selection pressure against the production of migrants. The anadromous population is more likely to include migrant and resident individuals leading to variation in developmental timing, and thus, more sex bias in gene expression. In addition, most anadromous populations of salmonids have a higher incidence of female migration than male migration. Therefore, differences in life history choice related to sex could also contribute to the elevated sex bias in gene expression observed in the anadromous population. All samples were raised in a common environment suggesting that observed differences in gene expression are the result of heritable differences between populations rather than influences from the environment, and therefore may be adaptive. Future research focusing on measuring sex bias in other populations of *O*. *mykiss*, especially those with similar resident and migrant populations, could be fruitful in determining how widespread sex bias in gene is in this species.

## Supporting information

S1 Table Raw data from all contrasts, component ID, raw and FDR corrected p values (alpha = 0.05) are reported. (XLSX)

S2 Table Annotated sex-biased genes. (XLSX)

S3 Table Biofunctional pathways that were altered between males and females in each of 10 contrasts. (XLSX)

S4 Table All canonical pathways altered between the sexes in at least one contrast. The Log_2_ p value for each pathway is reported.
(XLSX)

S5 Table Results from mapping differentially expressed genes to the *O. mykiss* genome. (XLSX)
